# Effects of medication intake on the risk of hemorrhage in patients with sporadic cerebral cavernous malformations

**DOI:** 10.3389/fneur.2022.1010170

**Published:** 2023-01-04

**Authors:** Bixia Chen, Kirstin Lahl, Dino Saban, Annika Lenkeit, Laurèl Rauschenbach, Alejandro N. Santos, Yan Li, Boerge Schmidt, Yuan Zhu, Ramazan Jabbarli, Karsten H. Wrede, Christoph Kleinschnitz, Ulrich Sure, Philipp Dammann

**Affiliations:** ^1^Department of Neurosurgery and Spine Surgery, University Hospital Essen, University of Duisburg-Essen, Essen, Germany; ^2^Institute of Diagnostic and Interventional Radiology and Neuroradiology, University Hospital Essen, University of Duisburg-Essen, Essen, Germany; ^3^Institute for Medical Informatics, Biometry and Epidemiology, University Hospital Essen, University of Duisburg-Essen, Essen, Germany; ^4^Department of Neurology, University Hospital Essen, University of Duisburg-Essen, Essen, Germany

**Keywords:** cavernous malformation, medication, intracerebral hemorrhage, hemorrhage risk, beta blocker, statin, antithrombotic therapy, thyroid hormone

## Abstract

**Objective:**

Recurrent intracerebral hemorrhage (ICH) poses a high risk for patients with cerebral cavernous malformations (CCMs). This study aimed to assess the influence of medication intake on hemorrhage risk in sporadic CCMs.

**Methods:**

From a database of 1,409 consecutive patients with CCM (2003–2021), subjects with sporadic CCMs and complete magnetic resonance imaging data were included. We evaluated the presence of ICH as a mode of presentation, the occurrence of ICH during follow-up, and medication intake, including beta blockers, statins, antithrombotic therapy, and thyroid hormones. The impact of medication intake on ICH at presentation was calculated using univariate and multivariate logistic regression with age and sex adjustment. The longitudinal cumulative 5-year risk for (re-)hemorrhage was analyzed using the Kaplan–Meier curves and the Cox regression analysis.

**Results:**

A total of 1116 patients with CCM were included. Logistic regression analysis showed a significant correlation (OR: 0.520, 95% CI: 0.284–0.951, *p* = 0.034) between antithrombotic therapy and ICH as a mode of presentation. Cox regression analysis revealed no significant correlation between medication intake and occurrence of (re-)hemorrhage (hazard ratios: betablockers 1.270 [95% CI: 0.703–2.293], statins 0.543 [95% CI: 0.194–1.526], antithrombotic therapy 0.507 [95% CI: 0.182–1.410], and thyroid hormones 0.834 [95% CI: 0.378–1.839]).

**Conclusion:**

In this observational study, antithrombotic treatment was associated with the tendency to a lower rate of ICH as a mode of presentation in a large cohort of patients with sporadic CCM. Intake of beta blockers, statins, and thyroid hormones had no effect on hemorrhage as a mode of presentation. During the 5-year follow-up period, none of the drugs affected the further risk of (re-)hemorrhage.

## Introduction

Cerebral cavernous malformations (CCMs) are rare vascular lesions accounting for ~5–10% of all cerebral vascular pathologies. Despite their benign nature, CCMs can lead to symptomatic intracerebral hemorrhages (ICHs) with an annual risk ranging between 2 and 6% (1–4). The major clinical symptoms are epileptic seizures and focal neurological deficits (4–6). Although the majority of cases show a benign course, recurrent hemorrhage in eloquent regions may result in high morbidity or even mortality in affected patients (7, 8).

In numerous studies, several CCM-related factors have been identified to affect the risk of hemorrhages, such as lesion localization, previous history of hemorrhage, and the presence of an associated developmental venous anomaly (2, 9–13). Among generic risk factors, obesity was shown to be associated with a higher risk of hemorrhage as a mode of presentation in patients with sporadic CCMs (14). By contrast, obesity appeared to have a protective effect in patients with familial CCM disease (15). In the recent past, there was evidence that several groups of drugs may have an impact on the bleeding risk of CCMs. Thus, it was suggested that the use of antithrombotic medications might reduce the risk of hemorrhage (16–18). In several anecdotal case series, propranolol intake led to lesion regression and cessation of recurrent bleeding in adult patients (19). Based on these findings, a randomized controlled pilot trial on the treatment of familial CCMs with propranolol was initiated (20), the results of which are awaited to be published. Statins are known to stabilize the cerebral vascular system by regulating the prenylation-dependent signaling pathway (21) and may have a positive effect on the course of CCMs (17, 22, 23). In addition, thyroid hormones were shown to affect the hemostatic system, whereby irregularities in the level of these hormones may lead to increased tendencies of bleeding (24, 25).

Further knowledge about the influence of medication intake on CCMs could substantially improve the current treatment strategies for this disease (26) as these are limited to either observation or invasive treatment (surgery/radiosurgery), the latter of which still carries the known significant intervention-related risks (27).

Importantly, this issue was addressed in the top 10 research priorities for CCMs that were just recently defined (28).

This study, therefore, aimed to examine the effect of medication intake on the risk of hemorrhage in sporadic CCMs in a large single-center observational cohort.

## Methods

From a patient registry with a total of 1,409 patients with CCMs who were consecutively treated in our institution between January 2003 and June 2021, 1,116 subjects with sporadic CCMs and available magnetic resonance imaging (MRI) data in our in-house picture archiving and communication system were included. Parts of the data from this patient cohort have been published before (10, 14). We evaluated the mode of presentation in all patients (prior symptomatic ICH [including cavernoma-related epilepsy (CRE)], non-hemorrhagic CRE, and asymptomatic) and occurrence of ICH during a 5-year follow-up period according to reporting standards (29) from the time of their first consultation in our clinic. We obtained baseline clinical data and medication status *via* a questionnaire. The follow-up data were acquired through routine examinations in our specialized outpatient clinic.

We searched our in-house picture archiving and communication system for the availability of a complete MRI protocol (T2, T1, T1, with CE, and T2^*^/susceptibility-weighted imaging) at the time of diagnosis and follow-up MRI. T1- and T2/T2^*^-weighted MR images were assessed for diagnosis, primary localization of CCM, and the presence of symptomatic ICH. Cases with spinal or familial CCMs (defined as a presentation with multiple CCMs without associated developmental venous anomaly, the occurrence of CCM in at least two family members, and/or positive genetic testing) and patients with incomplete MRI data were excluded.

The study was conducted according to the principles expressed in the Declaration of Helsinki. The protocol was approved by the local university Institutional Review Board (review board identification 14-5751-BO and 19-8662-BO). Written consent was obtained from all patients.

### Statistical analysis

Statistical analysis was performed using IBM SPSS Statistics Version 27.0.0.0 (SPSS Inc., IBM Corp., Armonk, New York, USA). Calculation of the association between medication intake and ICH as a mode of presentation was performed using binary logistic regression for each drug separately as the independent variable and with adjustments for age and sex as potential confounders. In addition, a multivariate logistic regression model was fitted, including all drugs with age and sex adjustments, as well as brainstem localization. Furthermore, the outcome during the 5-year follow-up period was assessed regarding the effect of medication intake on the occurrence of (re-)hemorrhage by performing a Cox regression analysis with adjustments for age, sex, and the presence of prior ICH. For the same time span, the Kaplan–Meier curves were plotted for the complete cohort and stratified by intake status for each medication. Data were censored when patients experienced (re-)hemorrhage, were subjected to invasive CCM treatment (operative or radiosurgery), or were lost to follow-up.

## Results

### Patient characteristics

According to the inclusion criteria, the data of 1,116 patients with sporadic CCMs were considered. The mean patient age was 42 years, ranging from 0 to 90 years. Of note, 683 patients (61.2%) were female, 507 patients (45.4%) initially presented with ICH, and 201 (18.0%) suffered from CRE. In 470 patients (42.1%), the CCM was asymptomatic. Intake of beta blockers was found in 8.0%, statins in 4.8%, antithrombotic therapy (including antiplatelet drugs and anticoagulants) in 8.9%, and thyroid hormones in 6.6% of the patients. The cohort's baseline characteristics are displayed in Table 1.

**Table 1:** Baseline characteristics of 1,116 patients with CCM.

**Demographics**	**Overall *n* = 1,116**
Mean age (years) (range, standard deviation)	42 (0–90, 15.9)
Female	683 (61.2%)
**Clinical presentation**	
Incidental	470 (42.1%)
Presentation with ICH	507 (45.4%)
CCM-related epilepsy^1^	201 (18.0%)
**Medication intake** ^**#**^	
Beta blockers (*n* = 826)	89 (8.0%)
Statins (*n* = 826)	54 (4.8%)
Antithrombotic therapy (*n* = 697)	62 (8.9%)
Thyroid hormones (*n* = 696)	74 (6.6%)
**CCM localization**	
Supratentorial	722 (64.7%)
Infratentorial non-brainstem	111 (9.9%)
Brainstem	283 (25.4%)

1 With or without associated ICH.

# *n* = number of complete data sets.

CCM, cerebral cavernous malformation; ICH, intracerebral hemorrhage.

### Medication intake and ICH as a mode of presentation

The logistic regression revealed a significant association between intake of antithrombotic medication and ICH as a mode of presentation in the single medication analysis with age and sex adjustments (OR: 0.520, 95% CI: 0.284–0.951, *p* = 0.034), indicating a lower chance of having ICH as a mode of presentation for patients taking antithrombotic medication. In the multivariate logistic regression model, including all drugs and age and sex adjustments, the OR for the association between antithrombotic therapy and ICH did not change (OR: 0.517, 95% CI: 0.255–1.047, *p* = 0.067). All results of the logistic regression analysis are shown in Table 2.

**Table 2:** Logistic regression analysis of medication intake and ICH as a mode of presentation (age and sex adjustments).

**Variable**	**Single medication analysis**	**All medication analysis**
	**OR (95% CI) *p*-value**	**OR (95% CI)** ***p*-value**
Beta blockers	1.226 (0.776–1.935) *p* = 0.382	0.683 (0.361–1.292) *p* = 0.241
Statins	1.226 (0.776–1.935) *p* = 0.465	1.273 (0.584–2.774) *p* = 0.543
Antithrombotic therapy	0.520 (0.284–0.951) *p* = 0.034	0.517 (0.255–1.047) *p* = 0.067
Thyroid hormones	1.126 (0.686–1.848) *p* = 0.640	1.046 (0.611–1.790) *p* = 0.871

### Cumulative 5-year outcome: (re-)hemorrhage during follow-up

For the assessment of (re-)hemorrhage risk during a 5-year follow-up period, a Kaplan–Meier analysis was performed. A total of 93 events occurred during 2642 person-years of follow-up, that is, 3.5 events per 100 person-years. The mean follow-up time was 28 months. In total, 294 patients (26.3%) underwent surgery and were censored due to the operation. The cumulative 5-year risk for symptomatic ICH was 17.6% (95% CI: 14.3–21.5%). From the group of patients without ICH at presentation, 1.5% experienced hemorrhage during follow-up. Within the group of patients with ICH as a mode of presentation, re-hemorrhage occurred in 23.3%.

**Figure 1:**
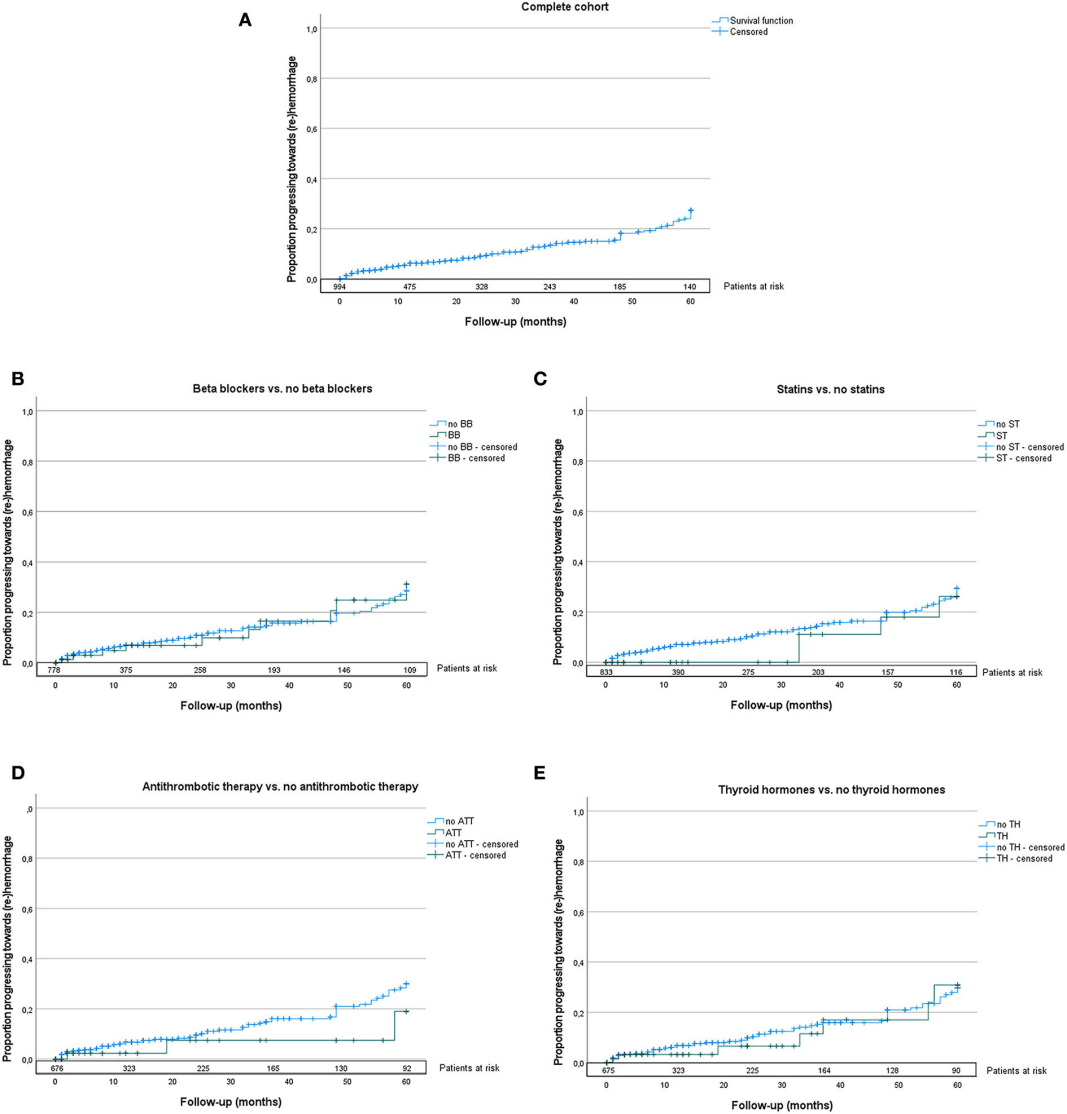
Kaplan–Meier curves of the complete cohort **(A)**, stratified by intake of beta blockers **(B)**, statins **(C)**, antithrombotic therapy **(D)**, and thyroid hormones **(E)** are shown; drugs are depicted in green. The numbers of patients at risk are marked on top of the *x*-axis of each graph. The log-rank test showed no significant differences between groups in the longitudinal analysis. BB, beta blockers; ST, statins; ATT, antithrombotic therapy; TH, thyroid hormones.

A cox regression analysis did not indicate strong associations between medication intake and the occurrence of (re)-hemorrhage, as shown in Table 3. Accordingly, the log-rank test for single-factor analyses revealed no significant risk differences for all drugs. Survival curves stratified by single medication intake are shown in Figure 1.

**Table 3:** Longitudinal outcome analysis: Cox regression analysis for medication intake and cumulative 5-year (re-)bleeding risk (age and sex adjustments).

**Variable**	**Cox regression analysis**	
	**Hazard ratio (95% CI)**	***p*-value**
Beta blockers	0.645 (0.310–1.342)	0.241
Statins	0.620 (0.249–1.546)	0.305
Antithrombotic therapy	0.937 (0.370–2.373)	0.892
Thyroid hormones	1.140 (0.484–2.686)	0.765

Further longitudinal single medication analyses adjusted for brainstem localization can be found in Supplementary material.

## Discussion

Few studies have assessed the effect of medication intake on CCM hemorrhage risk.

Although surgical resection of CCMs is curative, the operative treatment is associated with substantial perioperative risks, and only selected cases should be referred to surgery (26). Based on the consistently expanding application of MRI, an increasing number of incidental cavernomas are diagnosed. As this asymptomatic patient group is growing, conservative therapy with an observation of the lesion is usually conducted. Nevertheless, the assessment of risk factors for hemorrhage and the influence of potential medication on the disease in this patient collective is of high importance for further treatment. To this end, research addressing the impact of medication on the bleeding risk in patients with CCM was ranked among the top 10 priorities in CCM research (28).

This study assessed a set of medications that have a potential influence on hemorrhage tendency in a large cohort of patients with sporadic CCMs. We evaluated the mode of presentation and risk of (re-)hemorrhage during a 5-year follow-up period. The analysis of all parameters was conducted according to reporting standards (29). In our cohort, we found a significant mean reduction of ICH as a mode of presentation in patients with the intake of antithrombotic medication by 0.52 in the logistic regression analysis. In the multivariate analysis, including all drugs, the OR remained the same, with a trend toward statistical significance. All other drugs examined did not reach statistical significance regarding the prediction of ICH as the mode of presentation.

In the Cox regression analysis for 5-year follow-up, none of the medications reached statistical significance for the occurrence of (re-)hemorrhage. However, the results may have been biased due to the loss of some patients during the follow-up. Moreover, the group has a relatively high proportion of patients who underwent surgical treatment and whose data were subsequently censored; these factors may have caused over- or underestimation of hemorrhage events. Altogether, the limited completeness of follow-up and the relatively small number of patients with the intake of antithrombotic drugs may have led to biased effect size estimates in the Cox regression analysis.

When considering the potential pathomechanism underlying the possible protective effect of antithrombotic therapy, the role of acetylsalicylic acid (ASA) and cyclooxygenase-2 inhibitors within the inflammation cascade should be considered. They block endothelial cofactors that cause an increase in anticoagulant-activated protein C, which may lead to recurrent CCM hemorrhages (30). Animal experiments have revealed a general vascular protective effect of the two drug groups (31). In rodent models with rabbit elastase aneurysms, ASA intake prevented inflammation of the periadventitial tissue and vessel wall (32, 33). Comparable findings were obtained in humans, whereby ASA had the same beneficial influence on intracranial aneurysms, resulting in a lowered rupture risk (34–37). These aneurysm-related findings may also be valid for CCMs.

Our observations on antithrombotic therapy are in accordance with previous studies by Zuurbier (18), Flemming (16), and Gomez-Paz (17), who found a negative correlation between ASA intake and presentation with hemorrhage in their CCM cohorts.

The data of our collective showed no effects on the intake of beta blockers, statins, or thyroid hormones.

Zabramski and colleagues (19) reported about two cases with recurrent CCM hemorrhage with low-dose statin treatment; both patients showed regression of the lesion and no recurrent hemorrhage. The studies of Gomez-Paz (17) and Agosti (38) revealed less presentations with hemorrhagic events with statin intake than without. Currently, the ongoing randomized AT CASH EPOC trial (23) evaluates atorvastatin therapy in patients with CCM; results can be expected in a few years.

Regarding beta-blocker intake, Moschovi (39), Zabramski (19), and Reinhard (40) have published case reports with a striking benefit of propranolol treatment in patients with CCM. Lesions could be stabilized, and further hemorrhages were prevented by a propranolol dose of 20–60 mg/day. Given these promising results, the randomized Treat_CCM trial currently investigates the efficacy of this drug in the treatment of familial CCMs (20).

Our study has several limitations. The proportion of female patients (61%) was higher than that in other known larger studies [e.g., 58% [population-based] (41) and 54% [pooled-patient data] (2)]. Our study also included a high proportion of patients who initially presented with ICH (45%), although it was similar to other meta-analysis data (4). The medication status was not fully assessed in all patients. Moreover, the completion of follow-up limited longitudinal analyses of (re-)hemorrhage and may have masked a potential longitudinal effect. Medication intake was assessed based on self-reported questionnaires and medical charts, which may have led to over- or underreporting. Because of the observational nature of this study, unknown but confounding variables might have been neglected. Finally, the typical bias of a partially retrospective data collection may have been present in this study.

## Conclusion

In this cohort of adult patients with sporadic CCM, intake of antithrombotic therapy was associated with a lower risk of CCM hemorrhage as the mode of presentation. Intake of beta blockers, statins, and thyroid hormones did not affect the mode of presentation. During a 5-year follow-up, none of the medications affected the occurrence of (re-)hemorrhage.

## Data availability statement

The datasets presented in this study can be found in online repositories. The name of the repository and accession number can be found below: Dryad, https://datadryad.org/stash/dataset/doi:10.5061/dryad.9s4mw6mhs.

## Ethics statement

The studies involving human participants were reviewed and approved by Ethikkommission der Medizinischen Fakultät der Universität Duisburg Essen, identification 14-5751-BO and 19-8662-BO. Written informed consent to participate in this study was provided by the participants' legal guardian/next of kin.

## Author contributions

BC: conception, data acquisition, statistical calculations, and writing. PD: conception, data acquisition, calculations, proof reading, and supervision. US, CK, KW, and YL: proof reading. BS: proof reading and statistical calculations. AS, LR, AL, DS, and KL: proof reading and data acquisition. All authors contributed to the article and approved the submitted version.
