# Supplementary material for: Effects of medication intake on the risk of hemorrhage in patients with sporadic cerebral cavernous malformations
Source: Front Neurol. 2023 Jan 4;13:1010170. doi: 10.3389/fneur.2022.1010170 (PMC9847255; doi:10.3389/fneur.2022.1010170)
Supplement: Supplementary file 1 [file Table_1.docx]

**SUPPLEMENTAL MATERIAL**

Effects of medication intake on the risk of hemorrhage in patients with sporadic cerebral cavernous malformations

**Supplemental Table I**

Longitudinal outcome analysis: Cox regression for medication intake and cumulative 5-year (re) bleeding risk adjusted for brainstem localization.

| **Variable** | **Cox regression analysis**  **Single medication, adjusted for brainstem localization** | | **Cox regression analysis**  **All medication, adjusted for brainstem localization** | |
| --- | --- | --- | --- | --- |
|  | *Hazard ratio (95% CI)* | *p-value* | *Hazard ratio (95% CI)* | *p-value* |
| Beta blockers | 0.925 (0.476 – 1.798) | 0.818 | 1.140 (0.551 – 2.357) | 0.724 |
| Statins | 0.473 (0.171 – 1.308) | 0.149 | 0.457 (0.131 – 1.593) | 0.219 |
| Antithrombotic therapy | 0.467 (0.147 – 1.487) | 0.197 | 0.729 (0.214 – 2.486) | 0.614 |
| Thyroid hormones | 0.661 (0.300 – 1.460) | 0.306 | 0.684 (0.308 – 1.533) | 0.359 |
